# Information representation in an oscillating neural field model modulated by working memory signals

**DOI:** 10.3389/fncom.2023.1253234

**Published:** 2024-01-18

**Authors:** William H. Nesse, Kelsey L. Clark, Behrad Noudoost

**Affiliations:** ^1^Department of Mathematics, University of Utah, Salt Lake City, UT, United States; ^2^Department of Ophthalmology, University of Utah, Salt Lake City, UT, United States

**Keywords:** neural coding, phase, information theory, working memory, computational model

## Abstract

We study how stimulus information can be represented in the dynamical signatures of an oscillatory model of neural activity—a model whose activity can be modulated by input akin to signals involved in working memory (WM). We developed a neural field model, tuned near an oscillatory instability, in which the WM-like input can modulate the frequency and amplitude of the oscillation. Our neural field model has a spatial-like domain in which an input that preferentially targets a point—a stimulus feature—on the domain will induce feature-specific activity changes. These feature-specific activity changes affect both the mean rate of spikes and the relative timing of spiking activity to the global field oscillation—the phase of the spiking activity. From these two dynamical signatures, we define both a spike rate code and an oscillatory phase code. We assess the performance of these two codes to discriminate stimulus features using an information-theoretic analysis. We show that global WM input modulations can enhance phase code discrimination while simultaneously reducing rate code discrimination. Moreover, we find that the phase code performance is roughly two orders of magnitude larger than that of the rate code defined for the same model solutions. The results of our model have applications to sensory areas of the brain, to which prefrontal areas send inputs reflecting the content of WM. These WM inputs to sensory areas have been established to induce oscillatory changes similar to our model. Our model results suggest a mechanism by which WM signals may enhance sensory information represented in oscillatory activity beyond the comparatively weak representations based on the mean rate activity.

## Introduction

Signals from cortical areas reflecting cognitive states, including working memory (WM), have been shown to modulate responses to incoming sensory stimuli (Desimone and Duncan, 1995; Humphreys et al., 1998; Lee et al., 2005; Mitchell et al., 2007; Fries, 2009, 2015; Churchland et al., 2010; Bosman et al., 2012; Vinck et al., 2013; van Kerkoerle et al., 2014; Womelsdorf and Everling, 2015; Engel et al., 2016; Michalareas et al., 2016; Moore and Zirnsak, 2017). Several modeling studies have explored these effects (Brunel and Wang, 2001; Ardid et al., 2007; Lakatos et al., 2008; Kopell et al., 2011; Lee et al., 2013; Kanashiro et al., 2017). Firing rates of sensory cortical neurons are not modulated, or modulated only weakly, by WM alone (Lee et al., 2005; Zaksas and Pasternak, 2006; Mendoza-Halliday et al., 2014). In the presence of a sensory signal, firing rates of neurons in these sensory areas are enhanced when the stimulus in their receptive field is the target of WM (Merrikhi et al., 2018). However, these observed enhancements to firing rate are modest, and it is unknown how such small changes could account for the cognitive enhancements that WM produces (Bahmani et al., 2018). In addition to changes in mean activity, WM also induces changes to oscillations in local field potentials (LFPs) (Siegel et al., 2008; Liebe et al., 2012; Daume et al., 2017) and the timing of spikes relative to these oscillations (Lee et al., 2005; Bahmani et al., 2018). These oscillatory effects suggest a potential role of oscillatory phase of spikes in encoding information about a stimulus.

Prior results by our group (Bahmani et al., 2018) have shown that in a WM task, when a receptive field of an extrastriate visual neuron is in a remembered location, recordings show increased LFP oscillatory power and peak-power frequency in the α-β band (8–25 Hz), increasing spike phase locking (SPL) to the LFP oscillation. Stimuli present in the visual field also have been shown to increase LFP oscillatory power and peak-power frequency as a function of stimulus contrast (Roberts et al., 2013). Notably, cortical beta oscillations exhibit a diffuse relationship with individual spiking cells and show weak-to-moderate phase locking to the local field. Modeling work by our group (Nesse et al., 2021a) has also identified how WM signals to recurrent spiking networks incorporating excitatory (e) and inhibitory (i) cells could modulate emergent network oscillations and SPL, consistent with experimental findings in the study by Bahmani et al. (2018). While this prior modeling study identified the role of e- and i-cells coordinating these oscillatory effects, it suggest two further questions. First, is there a dynamical mechanism that gives rise to such oscillations and their modulation by WM signals? Second, does stimulus-driven changes to the timing of spike activity relative to the LFP phase serve as an effective means of encoding sensory information, and can it be enhanced by WM input?

To address these aforementioned questions, we devised an oscillatory mean field type or neural mass model (Wilson and Cowan, 1972) that exhibits oscillations. We find that the constellation of oscillatory effects due to WM (top down) input and stimulus (bottom up) input observed in previous studies (Roberts et al., 2013; Bahmani et al., 2018) are consistent with a dynamical system tuned near a supercritical Hopf instability, in which external input—either WM-like or sensory-like input–serves as a bifurcation parameter. Such instabilities give rise to low amplitude oscillations whose amplitude can be modulated by input, but whose amplitude is continuous with zero at the point of bifurcation.

Based on the hypothesis of supercritical Hopf instability, we identified a parameter regime in our model that exhibits such a tuning and verified that our neural mass model shows good correspondence with a large-*N* spiking model network. Using this parameter tuning, we then expanded our neural mass model to a neural field model by including a spatial-like domain, representing a stimulus feature parameter (Ben-Yishai et al., 1997; Shriki et al., 2003; Coombes et al., 2014). The activity of the neural field model can vary its responses to stimulus input features and can represent stimulus information in the mean rate response–the rate code—and the timing of activity relative to the phase of the global oscillation–the phase code. Using these two encodings, we then measured stimulus information coding performance of bottom-up inputs. We establish that the phase-based coding performance is *positively* enhanced by increased WM-like signals that increase oscillation amplitude and frequency, whereas the rate coding performance is reduced by the same WM input. Additionally, we show phase coding performance as a function of stimulus contrast that exhibits increased gain in the presence of WM input. Conversely, rate coding performance shows reduced stimulus contrast gain in the presence of WM input.

Furthermore, we find that phase code performance is roughly two orders of magnitude larger than the rate code. This large coding disparity can be explained by the nature of relaxation oscillations of local e-i network dynamics. The oscillations arise from the interplay between excitatory rising phase activity being curtailed by the delayed response of the inhibitory downward phase of the cycle. By definition, mean firing rate representations average over this oscillating cycle. As such, the adaptive inhibitory response partly obscures the information contained stimulus-induced activity, whereas the time course of e-i cycle of activity retains the underlying stimulus information more effectively (Nesse et al., 2021b).

## Methods

### Neural mass model

To assess sensory coding performance, we used the neural field model which was constructed from a spatial continuum of neural mass models connected through a spatial kernel. As such, we must first construct the neural mass model. We developed a neural mass model inspired by Wilson and Cowan (1972) representing a population-average activity of a recurrently connected network (i.e., a neural mass), in which *u*(*t*) and *v*(*t*) represent the mean synaptic activity of e- and i- cells, respectively.


(1) $$\begin{array}{l} \begin{array}{cc} \tau_{e}\frac{du}{dt}= & -u+f_{\sigma }\left(w_{ee}u-w_{ei}v+I_{e}\right),\\ \tau_{i}\frac{dv}{dt}= & -v+f_{\sigma }\left(w_{ie}u-w_{ii}v+I_{i}\right), \end{array} \end{array}$$

where the function *f*_*σ*_(*I*) in Eq. 1 is a “F-I” function representing the firing rate of a neural mass as a function of the average input current *I*. The F-I curve is parameterized by *σ* > 0 that controls the overall slope or firing rate gain, resulting from input current changes. The F-I curve is derived as the inverse of the mean first passage time of a leaky integrate and fire (LIF) model driven by input current *I* and uncorrelated noise with strength *σ* (see below). The *w*_*jk*_ is synaptic weight between and within cell types, and *I*_*j*_ is an offset current for *j, k* = *e, i*. We chose τ_*e*_ = 5 ms and τ_*i*_ = 15 ms monoexponential decay timescales, consistent with common estimates of effective excitatory AMPA receptor and inhibitory GABA_*A*_ decay timescales, respectively (Kapur et al., 1997; Xiang et al., 1998). We have chosen synaptic weights where the inter-population connections—*w*_*ei*_, *w*_*ie*_—were roughly double that of the intra-population connections—*w*_*ee*_, *w*_*ii*_ (see Table 1). This choice is consistent with the findings that e- and i-cell populations exhibit strong inhibition-mediated stabilization of the network (Sanzeni et al., 2020; Sadeh and Clopath, 2021). A complete list of parameter values is presented in Table 1.

**Table 1:** A list of parameter values of the neural mass and field models.

**Parameter**	**Value**
*V*_*t*_	−50 mV
*V*_*r*_	−65 mV
*V*_*l*_	−65 mV
*C*	1 mF
τ_*i*_	15 ms
τ_*e*_	5 ms
*g*	1/15 mS
*σ*_0_	5.5
*I*_*e*0_	−2.31
*I*_*i*0_	−3.81
Δ_*stim*_	0.018
Δ_*WM*_	0.015
*w*_*ee*_	0.9
*w*_*ii*_	1.9
*w*_*ie*_	1
*w*_*ei*_	2
κ	5.0625
κ_*s*_	20
*σ*_*y*_	0.02
τ_*y*_	50 ms

The neural mass model equilibria and stability of equilibria were computed over a wide parameter scan of input *I* and firing rate gain *σ* parameters. Both e- and i-cells received input controlled by a single *I* parameter, where *I*_*e*_ = *I* and *I*_*i*_ = *I*−0.5, where the inhibitory offset “−0.5” in *I*_*i*_ reflects higher spike thresholds for inhibitory cells that are typically used in neural mass models (Angelucci et al., 2017). Equilibria (u^*^, v^*^) were solved using a standard iterative Newton's method $\left(u_{n+1},v_{n+1}\right)=\left(u_{n},v_{n}\right)-{\text{J}}^{-1}\left(\left(u_{n},v_{n}\right)\right)\text{F}\left(\left(u_{n},v_{n}\right)\right)$, where **F** is the differential equation (DE) right hand side and **J** is the Jacobian matrix linearization of the DE. Stability of the equilibrium was obtained by computing the eigenvalues of the linearized model (i.e., the Jacobian matrix). Where Hopf instabilities were observed (see Results), we computed the first Lyapunov coefficient *ℓ*_1_ to determine the type of Hopf bifurcation—supercritical (*ℓ*_1_ < 0) or subcritical (*ℓ*_1_ > 0)—according to the standard formula (Kuznetzov, 1998).

### F-I curve derivation

The firing rate as a function of input current curve *f*_*σ*_(*I*) was derived from a LIF model stochastic differential equation (SDE) and subjected to white noise *σξ*(*t*) in which 〈*ξ*(*t*)〉 = 0 and 〈*ξ*(*t*)*ξ*(*t* + τ)〉 = δ_*t, t*+τ_ (Stein, 1965) are given by


(2) $$\begin{array}{l} C\frac{dV}{dt}=g\left(V_{l}-V\right)+I+\sigma \xi \left(t\right), \end{array}$$

with *V*(*t*) being the membrane voltage (mV); *C* = 1 the membrane capacitance in micro-Farads; *g* = 1/15 the leak conductance in micro Siemens; *V*_*l*_ = −65 mV is reversal leak potential; and *I* is the input current. With these parameters, a *I* = 1 nA of non-fluctuating input will induce the membrane to reach the spike threshold voltage *V*_*t*_ = −50. With the addition of fluctuating white noise input *σξ*(*t*) and assuming a reflecting boundary for very large negative voltages, the mean first passage time *T*(*V*; *I*, *σ*) for our LIF model (Eq. 2) to reach spiking threshold, while initially starting at *V*(0) = *V*_*r*_, solves the following differential equation with absorbing spike threshold boundary condition (Gardiner, 2009):


(3) $$\begin{array}{r} \begin{array}{r} \left(I+gV_{l}-gV\right)\frac{\partial T}{\partial V}+\frac{\sigma^{2}}{2}\frac{\partial^{2}T}{\partial V^{2}}=-1,\\ T\left(V_{t}:I,\sigma \right)=0. \end{array} \end{array}$$

The solution of (Eq. 3) is expressed as follows:


(4) $$\begin{array}{l} \psi \left(V\right)=e^{\frac{2V\left(I+gV_{l}\right)-gV^{2}}{\sigma^{2}}}, \end{array}$$

(5) $$\begin{array}{l} T\left(V;I,\sigma \right)=\frac{2}{\sigma^{2}}\int_{V}^{V_{t}}\psi {\left(s\right)}^{-1}\int_{-\infty }^{s}\psi \left(\xi \right)d\xi \;ds. \end{array}$$

The mean firing rate *f*_*σ*_(*I*) of the LIF model is then given by the inverse of the first passage time


(6) $$\begin{array}{l} f_{\sigma }\left(I\right)\equiv \frac{1,000}{T\left(V_{l};I,\sigma \right)}, \end{array}$$

expressed in units of Hertz.

The above mean first passage time is the spiking time of the LIF model in the diffusion limit of temporally uncorrelated synaptic δ-impulses (see Stein, 1965; Sanzeni et al., 2020).

### Obtaining the mean field model from a spiking LIF network

Considering a network of LIF model neurons as in Eq. 2, consisting of *j* = 1, 2, …, *N*_*e*_ and *ℓ* = 1, 2, …, *N*_*i*_ numbers of e- and i-cell constituents, each cell emits spikes at times *t*_*j, k*_ and *t*_*ℓ*, *k*_ for the spike timing index *k*, respectively. For simplicity, we assume that the network is all-to-all connected with uniform synaptic strength, in which the input current *I* to each e- and i-cell models is


$$\begin{array}{l} \begin{array}{c} I_{e}=I_{0e}+w_{ee}û\left(t\right)+w_{ei}\hat{v}\left(t\right),\\ I_{i}=I_{0i}+w_{ie}û\left(t\right)+w_{ii}\hat{v}\left(t\right) \end{array} \end{array}$$

respectively, where the synaptic currents û and $\hat{v}$ are given as follows:


(7) $$\begin{array}{l} \begin{array}{cc} \tau_{e}\frac{dû}{dt}= & -û+\frac{\Omega }{N_{e}}\overset{N_{e}}{\underset{j=1}{\sum }}\underset{k}{\sum }\delta \left(t-t_{j,k}\right),\\ \tau_{i}\frac{d\hat{v}}{dt}= & -\hat{v}+\frac{\Omega }{N_{i}}\overset{N_{i}}{\underset{\ell =1}{\sum }}\underset{k}{\sum }\delta \left(t-t_{\ell ,k}\right), \end{array} \end{array}$$

where δ is the Dirac delta functional and Ω is a synaptic strength parameter. We define the integral *Z* of either above sums in (Eq. 7) over a small time interval Δ*t* ≪ τ_*e*_, τ_*i*_ to be


(8) $$\begin{array}{l} \begin{array}{c} Z=\int_{t}^{t+\Delta t}\frac{\Omega }{N_{e}}\overset{N}{\underset{j=1}{\sum }}\underset{k}{\sum }\delta \left(s-t_{j,k}\right)ds. \end{array} \end{array}$$

In the limit of large *N*_*e*_ and *N*_*i*_, and the assumption of Poisson spike emission, the integral (Eq. 8) will have an expectation approaching the mean firing rate multiplied by Δ*t*, and the variance of the integral will limit to zero:


(9) $$\begin{array}{l} \begin{array}{l} \;\langle Z\rangle \sim f_{\sigma }\left(I\left(t\right)\right)\Delta t,\\ Var\left(Z\right)\sim 0. \end{array} \end{array}$$

Owing to Eq. 9, the sums in Eq. 7 are approximated by mean firing rates of the e- and i-cells, respectively, in which û → *u* and $\hat{v}\to v$ in probability. Therefore, Eq. 1 will approximate the mean of Eq. 7. Note that the synaptic strength can be set by the *w*_*jk*_ weights; so without loss of generality, we set the other strength parameter Ω = 1, 000 so that the numerical values of *u* and *v* track the mean firing rate, expressed in spikes per second (Hz).

Notably, in our model, for large *N*_*e*_ and *N*_*i*_, the variability of Eq. 7 limits to zero, and consequently, the external Weiner noise input *σξ*(*t*)*dt* accounts for all of the noisy fluctuating input to each cell. We choose this limiting case so that we can study emergent oscillatory dynamics by retaining the standard deviation *σ* of the noise as a control parameter. This limiting case contrasts with a different limiting case, in which, in the absence of any external fluctuating input, a recurrent neuronal network can self-generate its own variability *via* deterministic chaos. These networks are characterized by variance and covariance order parameters, whose values are determined by self-consistency conditions in which mean and (co)variability of input of each cell must match its output (Moreno-Bote et al., 2008; di Volo et al., 2019; Sanzeni et al., 2020). We have chosen our approach, in which input fluctuations are not enforced to be self-consistent with output fluctuations of network elements to allow flexibility to assume that input fluctuations may be dictated by external inputs, and in addition, we can use the noise parameter *σ*, an exploratory tuning parameter, to discover possible regimes that support oscillatory solutions.

We simulated networks of *N*_*e*_, *N*_*i*_ = 20, 000 cells to verify that the spiking network model's population firing rates are a decent match to that of the mean field model over 10-s simulations. We defined the LFP proxy of the network as the average of all currents to excitatory neurons *I*_*LFP*_(*t*) = *w*_*ee*_*u*(*t*) − *w*_*ei*_*v*(*t*) (Mazzoni et al., 2015).

### Neural field model

The neural field model is a neural mass model extended over a spatial domain (Coombes et al., 2014), in which nearby regions on the spatial domain are preferentially connected. We have chosen a ring topology spatial domain, parameterized by θ ∈ [0, π), which is intended to represent a hypercolumn-like population, with each *θ*-value referring to sub-population of the neural field that is selectively responsive to a *θ*-parameterized stimulus feature akin to a familiar bar visual stimulus with orientation angle *θ* (Ben-Yishai et al., 1997; Shriki et al., 2003). The neural field model is described by *u*(*θ*, *t*), *v*(*θ*, *t*), the e-activity and i-activity for every *θ* location on the ring, that solves the integro-differential equation as follows:


(10) $$\begin{array}{l} \begin{array}{c} \tau_{e}\frac{\partial u}{\partial t}=-u+f_{\sigma }\left(W*\left[w_{ee}u-w_{ei}v\right]+I_{e}\right)\\ \tau_{i}\frac{\partial v}{\partial t}=-v+f_{\sigma }\left(W*\left[w_{ie}u-w_{ii}v\right]+I_{i}\right). \end{array} \end{array}$$

The integral convolution “*” in Eq. 10 is over the *θ*-domain—$W*h=\int_{0}^{\pi }W\left(\theta -\theta^{\prime }\right)h\left(\theta^{\prime }\right)d\theta^{\prime }$, for any *h*—and the 𝒲(*θ*) von-Mises periodic weight kernel:


(11) $$\begin{array}{l} W\left(\theta \right)=\frac{e^{\kappa \cos\left(2\theta \right)}}{\pi I_{0}\left(\kappa \right)}. \end{array}$$

With this weight kernel (Eq. 11), two points on the ring at *θ* and *θ*′ will be connected with weight 𝒲(*θ*−*θ*′)*d*θ**. The parameter κ is an inverse variance-like scale parameter, where increasing κ produces a narrower distribution, and *I*_0_(κ) is the order-zero modified Bessel function of the first kind, which serves as the normalization constant. We chose the spatial scale of connection to be sufficiently broad (see Figure 3A top left sub panel) so as to support spatial stability across the ring. Moreover, the same spatial scale κ was used for e- and i-cell populations which also typically result in spatial stability across the ring (see Ali et al., 2016), giving rise to, for example, a uniform bulk oscillation (as in Figure 3A). Having a broader distribution footprint for e- relative to i-cell connection or a narrower overall scale for each subpopulation (larger κ) is typically associated with spatial instabilities (Coombes et al., 2014).

Stimulus inputs are orientation-tuned, given similarly by a Von-Mises-like distribution function


(12) $$\begin{array}{l} S\left(\theta \right)=e^{\kappa_{s}\cos\left(2\left(\theta -\theta_{0}\right)\right)-\kappa_{s}}, \end{array}$$

in which the peak strength (set to unity) of the stimulus located at orientation *θ*_0_ = π/2 and the input spread set by κ_*s*_ (see red curve, Figure 3A bottom left sub panel).

The LFP proxy is defined as the α-β-band filtered e-cell total input current (10–30 Hz notch filter), averaged over the ring *I*_*e, tot*_(*t*) = 〈𝒲*[_*w*_*ee*_*u*−*w*_*ei*_*v*]+*I*_*e*_〉*θ*_ (see Mazzoni et al., 2015), where 〈·〉_*θ*_ is the ring average. The phase ϕ(*t*) of this resulting oscillating signal is given by computing the Hilbert transform 𝕳 of *I*_*e, tot*_(*t*)


(13) $$\begin{array}{l} 𝕳\left(I_{e,tot}\left(t\right)\right)=I_{e,tot}\left(\theta ,t\right)+iA\left(\theta ,t\right), \end{array}$$

and then taking the angle argument of the resulting analytic signal:


(14) $$\begin{array}{l} \phi \left(t\right)\equiv Arg\left(I_{e,tot}+iA\right)\left(t\right). \end{array}$$

The phase has an inverse function we defined to be $\tilde{g}\left(\phi \right)\equiv t$.

On simulations that were analyzed to assess information coding performance, we included slow-timescale Ornstein-Ulenbeck noise *y*(*t*) to both e- and i-cell input currents *I*_*e*_ and *I*_*i*_ globally to the entire network (uniformly across all *θ*-values). While the origins of the oscillatory dynamics in our model arises from a deterministic Hopf instability, the purpose of this additional noise was to introduce variability in the oscillation amplitude and period, such as those observed in real cortical tissues, as well as to sample oscillatory dynamics for a fluctuating input near the oscillation threshold. However, we have not chosen to implement independent noise inputs across the neural field domain (non-uniform input). Such non-uniform noise would naturally lead to degraded coding performance for both phase and rate representations of activity. The relative degree of degradation for the two codes is an important question but is not addressed in this article. The dynamics of *y* are given by the SDE


(15) $$\begin{array}{l} \tau_{y}\frac{dy}{dt}=-y+\sigma_{y}\sqrt{\tau_{y}}\xi \left(t\right), \end{array}$$

where *ξ*(*t*) is uncorrelated zero-mean, unit-variance gaussian white noise. This SDE (Eq. 15) results in a normal stationary distribution of currents *q* with zero-mean, standard deviation *σ*_*y*_, and a temporal autocorrelation decay timescale τ_*y*_ = 50 ms so that the network oscillations, which were typically in the ~20 Hz β-band range (~50 ms cycles), showed robust cycle to cycle variability but little long-timescale multi-cycle correlation.

We wish to study how input modulations akin to WM signals can modulate the response properties—both rate responses and oscillatory responses—of neural masses within the neural field. In the absence of any external input, the neural field model parameters were set to a point near a supercritical Hopf instability where oscillations were small-amplitude, with an oscillation frequency in the β-band approximately 15–20 Hz. We ran simulations from this set point over four levels of a global WM input and four levels of orientation-selective stimulus input, starting from zero, which models the effective contrast of the stimulus. We call these WM 0, 1, 2, and 3 levels and stimulus contrast levels 0, 1, 2, and 3. Altogether, the input to cells can be represented by


(16) $$\begin{array}{l} I_{e}\left(\theta ,t\right)=I_{e0}+a\Delta_{stim}S\left(\theta \right)+b\Delta_{WM}+y\left(t\right)\\ I_{i}\left(\theta ,t\right)=I_{i0}+a\Delta_{stim}S\left(\theta \right)+b\Delta_{WM}+y\left(t\right), \end{array}$$

where Δ_*stim*_ and Δ_*WM*_ are the current increments for the respective input levels of stimulus *a* = 0, 1, 2, 3 and WM *b* = 0, 1, 2, 3. For the neural field simulations, we fixed the noise parameter to *σ* = 5.5. The complete list of parameter values of the neural mass and field models are given in Table 1.

### Information-theoretic measures of phase and rate coding

We wanted to test the ability of the neural field ring model to represent distinct sensory stimuli, so we tested the discriminability of the neural field activity across distinct stimuli positions on the ring. We gave elevated input centered at *θ*_0_ = π/2 according to Eq. 12 and assigned this to be the favored stimulus region, whereas elsewhere were less stimulated regions.

Each oscillation cycle is characterized by a phase decomposition ϕ(*t*) of the LFP proxy (see above) in which ϕ ∈ −π, π]. A typical cell at a location *θ* fires at the instantaneous mean rate described by the F-I curve *f*_*σ*_(*I*(*θ*, *t*)) as a function of the input current *I*(*θ*, *t*). Spikes are distributed over the oscillation cycle period *T* in proportion to the instantaneous firing rate *f*_*σ*_(*I*(*θ*, *t*)). To obtain a phase distribution from the instantaneous firing rate over the cycle period, we performed a change of variables from the time domain to the phase domain given by the function $\tilde{g}\left(\phi \right)=t$ (see Eqs. 13, 14). The phase density obtained from this change of variables is


(17) $$p_{\theta }(\phi )=\frac{f_{\sigma }(I(\theta ,\tilde{g}(\phi ))}{\int_{0}^{T}f_{\sigma }(I(\theta ,t))dt}\frac{d\tilde{g}}{d\phi }(\phi ),$$

where $\frac{d\tilde{g}}{d\phi }\left(\phi \right)$ is the change-of-variables Jacobian factor, and the integral in the denominator is the normalization ensuring $\int_{-\pi }^{\pi }p_{\theta }\left(\phi \right)d\phi =1$. We assume the typical cell at a location *θ* emits a Poisson-distributed number of spikes per unit time, with poisson parameter $\lambda_{\theta }\equiv T^{-1}{\langle f_{\sigma }\left(I\left(\theta ,t\right)\right)\rangle }_{T}$, where 〈·〉 represents a temporal average over a cycle of time-length *T*. Analogously, we define spikes per cycle as ${\hat{\lambda }}_{\theta }=\lambda_{\theta }T$ to be emitted by a typical cell, yielding a discrete Poisson distribution for the number of spikes *n* in the same oscillation cycle window *T*: $r_{\theta }\left(n\right)=\frac{{\hat{\lambda }}_{\theta }^{n}e^{-{\hat{\lambda }}_{\theta }}}{n!}$.

We computed two measures of coding performance. The first examines the discriminability of two *θ*-stimuli points on the ring. Without loss of generality, we chose *θ* = π/2, π/4 for our two representative points. The discriminable information or information gain (IG) is defined as the Kullback–Libler divergence (in nats) $D_{KL}\left(q_{\pi /2}||q_{\pi /4}\right)=\underset{z}{\sum }p\left(z\right)\ln\;\left(q_{\pi /2}\left(z\right)/q_{\pi /4}\left(z\right)\right)$, where *z* = ϕ and *q* = *p* for the phase code and *z* = *n* and *q* = *r* for the rate code:


(18) $$\begin{array}{l} \begin{array}{c} IG_{\phi }\left(\pi /4\to \pi /2\right)=D_{KL}\left(p_{\pi /2}||p_{\pi /4}\right)\\ IG_{r}\left(\pi /4\to \pi /2\right)=D_{KL}\left(r_{\pi /2}||r_{\pi /4}\right). \end{array} \end{array}$$

The IG (Eq. 18) measures the amount of information learned about the data distributed according to *p*_π/2_ given one scores data according to *p*_π/4_, minus the true entropy of the data.

The second measure of coding performance we computed was the mutual information (MI) between stimulus feature *θ* and either the oscillation phase variable ϕ or the spike count rate variable *n*. For concreteness, we chose to represent the stimulus feature across eight points, which evenly distributed about the ring $\theta_{k}=\frac{\pi k}{8}$, for *k* = 1, 2, …8. We assume on a “trial” that each of the eight stimuli was equally likely to occur, in which case, the probability of each stimuli was 1/8. The MI then can be constructed to be the Kullback–Libler divergence from the joint phase distribution $p_{\theta_{k}}=\frac{1}{8}\overset{8}{\underset{j=1}{\sum }}p_{\theta_{j}}\left(\phi \right)\delta_{jk}$ (where δ_*jk*_ is the Kronecker delta) to the marginal distribution over the *θ*_*k*_ stimuli: $\bar{p}\left(\phi \right)=\frac{1}{8}\overset{8}{\underset{j=1}{\sum }}p_{\theta_{j}}\left(\phi \right)$ and similarly for the rate code. This formula can be generalized to any number of points on the ring. For each code, the MI is also expressible in terms of the entropy ℋ of the average phase (rate) code across the stimuli, minus the average entropy of each phase (rate) code at a given stimulus point:


(19) $$\begin{array}{l} \begin{array}{c} MI_{\phi }\left(\pi /2;\pi /4\right)=D_{KL}\left(p_{\theta_{k}}||\bar{p}\right)=H\left(\bar{p}\right)-\frac{1}{n}\overset{8}{\underset{k=1}{\sum }}H\left(p_{\theta_{k}}\right)\\ MI_{r}\left(\pi /2;\pi /4\right)=D_{KL}\left(r_{\theta_{k}}||\bar{r}\right)=H\left(\bar{r}\right)-\frac{1}{8}\overset{8}{\underset{k=1}{\sum }}H\left(r_{\theta_{k}}\right). \end{array} \end{array}$$

We simulated the neural field over a 10-s duration time window over each of the 16 WM and stimulus input value combinations. For each simulation, using the notch filtered LFP (10–30 Hz), we segmented the LFP into individual cycles with individual periods. On each cycle, we computed phase and rate encoding distributions and computed the aforementioned information performance measures *IG* and *MI* and examined the average and variability across cycle trials and conditions.

### Numerical methods

All numerical simulations and calculations were performed using custom-written Matlab (Natick, MA) code. Simulations of DE models utilized a fixed step time of Δ*t* = 0.02 ms. Simulations of the two-variable neural mass model without noise were performed using a standard Matlab “ode23” DE solver. Notably, the F-I curve we used involved solving the mean first passage time (Eqs. 4, 5) that consisted of nested integrals, which would be numerically inefficient to function-call on each time step. Instead, we computed these curves once over a fine mesh and used a cubic spline fit in lieu of the exact solution.

Stochastic simulations of the LIF spiking model network model were computed using the Euler–Maruyama method, and an explicit 2nd-order Heun's method was used for all deterministic variables, with Δ*t* = 0.02

The neural field model was simulated by partitioning the orientation tuning domain in 360 equally spaced grid points along the *θ*∈[0, π) domain. An explicit 2nd-order Heun's method was used for all deterministic variables, while noise input was computed each using a first-order explicit Euler–Maruyama method (Gardiner, 2009).

## Results

### Analysis of neural mass model

The main question we address in this article is how oscillatory and non-oscillatory sensory representations in neural populations can be modulated by top-down input. In order to do so, we first investigate a e- and i-cell recurrent network that supports oscillations in isolation, termed the neural mass model. Then, in the following section we construct a neural field model consisting of many of these e- and i-cell subpopulations that will support sensory coding.

**Figure 1:**
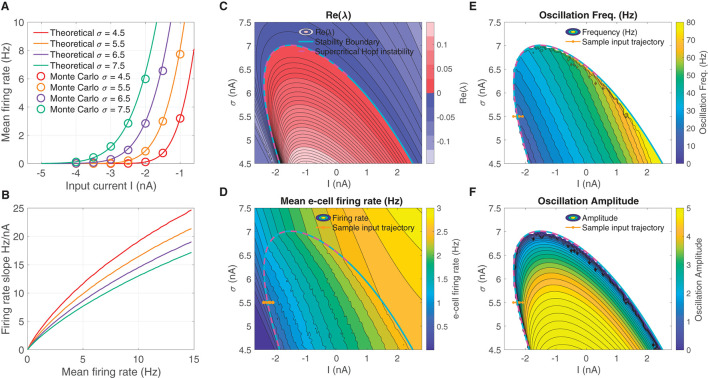
An unstable equilibrium, in which oscillations emerge, occurs in a protruding region in *I*-*σ* parameter space. **(A)** F-I curve solutions *f*_*σ*_(*I*) to Eqs. (4–6), parameterized by the diffusion noise *σ* parameter. **(B)** Increasing *σ* reduces the overall firing rate gain at a given firing rate *df*_*σ*_/*dI*|_*f*_*σ*__. **(C)** The real part of the smallest-magnitude eigenvalue Re(λ_*k*_) parameterized by the input *I* (abscissa), and F-I curve gain parameter *σ* (ordinate). There is a subregion where the eigenvalues are complex ω = Im(λ_*k*_) ≠ 0, in which crossing into the instability region produces a supercritical Hopf bifurcation (magenta dashed line). **(D)** Moving from left to right and from low, to high, the mean firing rate increases in a continuous manner, both outside and inside the unstable region. **(E)** Inside the unstable region, the oscillation frequency increases going diagonally up and to the right. **(F)** The supercritical Hopf bifurcation elicits oscillation amplitudes that emerge continuously from zero on the upper part of the unstable region. In **(D–F)** the brown line from left to right indicates a input parameter path of interest where oscillations emerge via supercritical Hopf in which mean firing rate, oscillation frequency, and weak amplitude oscillations increase continuously from zero.

We derived a mean field model of these spiking networks characterized by a 2D nonlinear system of DEs (Eq. 1) that describe the mean activity of the e- and i-cell populations, in which the mean firing rate is dictated by a firing rate function—the F-I curve—as a function of input current. The F-I curve *f*_*σ*_(*I*) was defined as the firing rate of an LIF model cell subject to noise input with strength *σ* (see Eqs. 4–6), and are shown in Figure 1A for several *σ*-values. The curves show good correspondence to the firing rates obtained from Monte Carlo simulations of the LIF cells. Increasing *σ* increases firing rates overall, resulting in an leftward translation of the curves. In addition, increasing *σ* has the effect of reducing the firing rate gain $\frac{df_{\sigma }}{dI}$ at any fixed firing rate *f*_*σ*_ (Figure 1B; see for instance Chance et al., 2002; Ferguson and Cardin, 2020).

The we searched for oscillatory instabilities in our neural mass model (Eq. 1) by performing a wide parameter scan over *I*-*σ* combinations. For each *I*-*σ* combination, we located equilibria and computed linear stability. Figure 1C shows there is an unstable region in *I*-*σ*-space where an eigen-analysis returns positive real part eigenvalues of the linearized system. Parameter pairs (*I*, *σ*) that move upward to the right produce increased mean e-cell firing rate (Figure 1D). Entering the left-side of the unstable region in *I*-*σ*-space from left to right, yields a supercritical Hopf bifurcation (see magenta-dashed line, where the Lyapunov coefficient is negative). Moving left to right through the bifurcation (see example brown-line trajectory) also results in oscillations with frequencies in the β-range 12–30 Hz (Figure 1E), whose amplitudes increase continuously from zero (Figure 1F).

**Figure 2:**
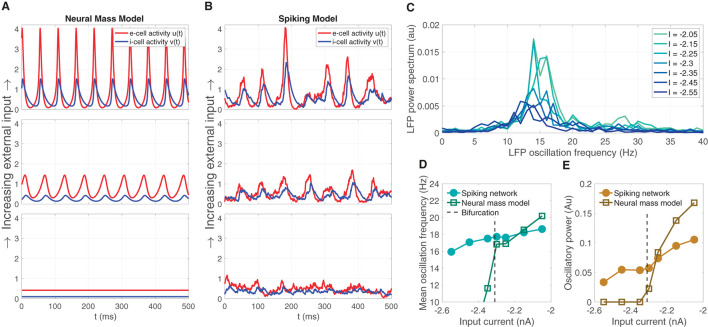
The emergence of oscillations through increased input in the neural mass model corresponds similar emergent oscillations in the large-*N* LIF spiking network model from which the neural mass model was derived. **(A)** Sample solution trajectories of the neural mass model at three ascending input levels (*I* = −2.45, −2.3, −2.15) one below the bifurcation (lower panel), one just slightly above (middle), and one further beyond (top). **(B)** The large-*N* (*N*_*e*_, *N*_*i*_ = 20, 000) LIF spiking network driven at the same inputs and parameter settings as the neural mass model in **(A)**. **(C)** The power spectrum of the large-*N* LIF model over seven input levels (four more levels interleaved between the three illustrated in **(A, B)** shows that increasing input *I* produces greater oscillatory power, and a shift toward higher frequencies within the *β*-oscillatory band. **(D, E)** The mean oscillation frequency and oscillatory power, respectively, computed from the power spectra in **(C)** for the LIF spiking network, and the neural mass model.

The neural mass model is was derived as a simplified representation of the dynamics of a large-*N* spiking model consisting of excitatory and inhibitory LIF model neurons. The mean field model predicts that oscillations can emerge from increasing mean input *I* (for a fixed *σ* = 5.5), depicted by the brown line in Figure 1F. Sample solutions of the neural mass model are shown for three input levels (*I* = −2.45, −2.3, −2.15) in Figure 2A, one below the bifurcation where a equilibrium solution is stable, one slightly past the bifurcation point where small-amplitude oscillations emerge, and one further past the bifurcation where larger-amplitude oscillations are observed. Using the same input levels and shared parameter settings, we also simulated a LIF spiking network model (*N*_*e*_, *N*_*i*_ = 20, 000). These stochastic simulations show a correspondence to the neural mass model solutions. At the lower input level, weak fluctuations exist, reflecting the fluctuation-driven spiking in the finite-*N* networks. At the predicted point of the neural field bifurcation, the LIF network exhibits emergence of more regular, intermediate-amplitude oscillations; while increasing input further leads to larger amplitude oscillations, in a manner similar to the neural mass model.

Supercritical Hopf bifurcations yield oscillation magnitudes that are continuous with zero at the point of bifurcation. Commensurate with the mean field model, we expect that the large-*N* LIF spiking network exhibits LFP power spectra that show an increase in oscillatory power with input. We also expect the central peak of LFP power to shift to higher frequencies as input is increased through the predicted bifurcation point. Figure 2C shows this predicted increase oscillation power and shift in frequency, both centered in the β-band, over seven input levels (we interleaved four more input levels between the three depicted in Figure 2B). This predicted increase in mean LFP oscillation frequency and oscillatory power in the LIF model network is summarized in Figures 2D, E, respectively, and shows a strong correspondence to those computed for the neural mass model. Note, however, that the LIF network model exhibits non zero power for input levels below the bifurcation point; whereas the neural mass model oscillatory power is necessarily zero. This is naturally due to the LIF network being a stochastic simulation in which case we expect small fluctuations. Such sub-threshold transient oscillatory behavior has been suggested to endow networks with useful function (Palmigiano et al., 2017). Importantly, just above the bifurcation point, there is good correspondence between the oscillatory frequency and power of the neural mass and LIF spiking model.

### Phase and rate representations of stimulus inputs in the neural field model

**Figure 3:**
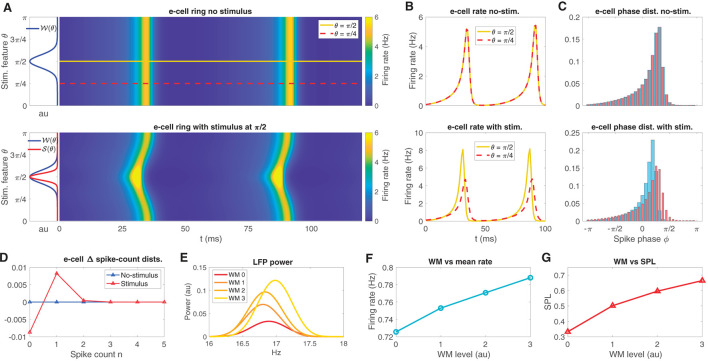
The neural field model replicates oscillatory behaviors observed in sensory areas during WM tasks. **(A)** The neural field e-cell oscillatory dynamics over its feature space in the presence of no stimulus (top), depicted along with the weight kernel 𝒲 (top left sub panel; see Eq. 11). In the presence of a stimulus 𝒮 (bottom, left sub panel red curve; see Eq. 12), the e-cells exhibit an oscillation with a bend located at the stimulus location. Two example stimulus feature locations *θ* = π/2, π/4 are shown on the ring in the top panel, yellow, red-dash, respectively. Neural field model example simulations exhibit phase locked oscillations across the ring, but preferred stimulus exhibits earlier activity. **(B)** e-cell firing rate on ring at the two example locations over 100ms simulation time for increasing stimulus contrast from no-stimulus (top) and with-stimulus (bottom). **(C)** The associated spike phase distributions for the same two example stimulus features as in **(B)** in no-stimulus and with-stimulus (top and bottom). **(D)** The difference in spike count distributions between the two example stimulus features in no-stimulus and with-stimulus conditions shows weak (~ 10^−3^) average spike count changes due to stimulus, while peak firing rate during an oscillatory cycle exhibits 3Hz differences between stimuli [see **(B)**, bottom]. **(E–G)** Increases in WM input produce LFP peak power and LFP frequency increases, mean firing rate increases, and increased in SPL, respectively.

The neural field model is a spatially extended neural mass model (see Coombes et al., 2014) with a ring connection topology, designed to represent an orientation tuning domain in which nearby regions on the ring are preferentially connected. We tuned each point on the ring to be near the supercritical Hopf instability that we identified in the neural mass model. In the absence of any *θ*-dependent input, the neural field exhibits a bulk oscillation (Figure 3A top panel; see also Mosheiff et al., 2023) due to the lateral connections in the network producing oscillation syncrony across the ring. The synaptic weight function 𝒲(*θ*) (see Eq. 11, centered at π/2 for convenience) is also depicted in Figure 3A (left sub panel of top panel). Three types of external inputs were given to the neural field: stimulus-like inputs, WM-like inputs, and temporally fluctuating random inputs (see Eqs. 15, 16).

Stimulus-like inputs are tuned to preferentially activate one *θ*-tuned region of the ring over other regions. These *θ*-tuned stimulus inputs induce changes to the time course of the bulk oscillation as well as mean firing rates across the ring. For example, Figure 3A (bottom panel) shows e-cell activity with the π/2-tuned input producing a temporal bend in the oscillation across the ring arising from elevated input in regions centered about *θ* = π/2. The synaptic input function 𝒮(*θ*) (Eq. 12) is depicted along with 𝒲(*θ*) in Figure 3A (left sub panel of bottom panel). This input induced the oscillation to initiate sooner in regions near π/2 relative to elsewhere on the ring.

To illustrate the effect of featured tuned inputs on the relative timing of oscillatory activity across the ring, we chose two stimulus features on the ring, a favored feature *θ* = π/2 from which we discriminate from the unfavored feature *θ* = π/4 (see Figure 3 top panel, yellow, red-dash, respectively), and from which we have defined a two-point discrimination task (see below). At these two positions on the ring, the mean instantaneous firing rate curves *f*_*σ*_(*I*(*t*)) are shown in Figure 3B for no-stimulus (top panel) in which the two features exhibit the exact same rate dynamics as the bulk oscillation. When stimulus centered at *θ* = π/2 is given, the firing rate of the favored feature exhibits a larger amplitude peak that occurs earlier than peak at the unfavored location (bottom panel).

The oscillatory phase angle of the neural field is defined by the phase decomposition ϕ(*t*) of the LFP proxy signal (via Hilbert transform, see Methods). Here, LFP is defined as the mean e-cell input over the entire ring (Mazzoni et al., 2015). Figure 3C shows the phase-distributions at the *θ* = π/2 and π/4 locations (see Eq. 18). Consistent with the firing rate time courses (in Figure 3B) the phase distributions reflect that without stimulus input, the phase distributions are identical (top). A stimulus input at *θ* = π/2 produces more spikes to occur earlier in the neural field model's oscillatory cycle and is more sharply peaked than the phase distribution at the unfavored feature *θ* = π/4.

Note that while the phase signatures of the stimulus are salient to the eye in Figure 3C, the changes to the average firing rate over the cycle are very weak. Figure 3D shows the differences in the spike count distributions that defines the rate code in the stimulus and no-stimulus conditions. Naturally, the no-stimulus case has zero difference between spike count distributions; however, even while the peak firing rate during the oscillation cycle differs by about 3 Hz between the favored and unfavored locations (see Figure 3B bottom), the spike count distributions over the entire cycle differ by at most ~1 × 10^−2^, which is small relative to the phase distribution differences.

In contrast with stimulus inputs, WM-like inputs are global, uniform over the ring. Increases to global WM input (with WM input random fluctuations, see Methods) to the neural field ring produces increases in LFP peak power and frequency in the beta band (Figure 3E). Finally, WM input has a positive effect on mean firing rate of the ring (Figure 3F), and mean spike phase locking (SPL; Figure 3G; SPL is 1 minus the circular variance). These effects observed in the neural field model are consistent with the constituent neural mass model, and are also consistent with experimentally observed WM-driven changes observed in extrastriate areas (Bahmani et al., 2018).

Finally, temporally fluctuating random inputs were included to simulations that we used to perform our sensory coding analyses (see below). We included these noise inputs to elicit a broad sampling of oscillatory frequencies and amplitudes, particularly near the deterministic bifurcation.

### Sensory coding performance of phase and rate codes

We sought a rigorous way to discriminate representations of activity between stimulus features on the ring *θ* by representing the neural field model solution dynamics based on two coding schemes: an oscillatory code, and a spike rate code. Specifically, we wanted to determine how stimulus discrimination performance was affected by stimulus contrast level as well as the WM-input levels. For both of the coding schemes, we computed two measures of coding performance. First, we computed the IG [see Methods (Eq. 18), also termed the relative entropy or Kullback-Libler divergence], measuring the discrimination information between two representative points on the ring: *θ* = π/2 and π/4. Second, we computed MI (Eq. 19) between the stimulus ring location variable *θ* and the neural activity representation variable—either phase or rate representations. We computed these measures over each oscillation cycle of the model-generated data from 10 s of simulation, to generate averages and standard deviations for each performance measure.

**Figure 4:**
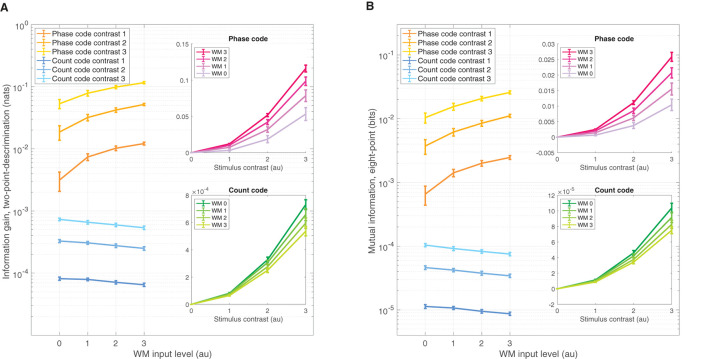
Divergent sensory discrimination performance is exhibited by the phase and rate codes as a function of WM input. **(A)** IG measure shows enhancement for the phase code across all non-zero stimulus contrasts while the rate code is reduced, measured on a log ordinate scale. Insets of **(A)** The same data as the main panel but plotted on a ordinate linear scale as a function of contrast, which shows increasing WM input enhances the coding gain as a function of contrast while reducing rate coding gain for the same WM increases. **(B)** Qualitatively similar results to **(A)** but for the MI coding performance measure. All error bars reflect standard deviations.

The IG is a simple means of assessing amount of information one gains from observing data obtained from the π/2-parameterized distribution if one had a initially assumed the data came from the π/4-parameterized source. Greater IG underlies greater statistical discrimination power from which one can formulate a decision threshold to detect the π/2 stimulus over π/4. Figure 4A shows the IG measure for both the phase code (warm colors) and spike rate code (cooler colors) on ordinate log scale. Naturally, increasing contrast improves performance across all WM-input levels; although the zero contrast discrimination performance was, of course, zero, and was not shown on the log scale. Interestingly, WM-input increases produce positive enhancements in the phase code across all non zero contrast levels. Conversely, the spike rate code shows the opposite effect—a reduction of coding performance results from WM input increases. The insets show the same data as a function of contrast level in linear ordinate scale, which reveals that increasing WM input enhances stimulus contrast gain in the phase code, while simultaneously reducing the spike rate code gain. Note also that the phase code over all contrasts and WM levels was about two orders of magnitude larger than that of the spike rate code. These effects were significant: over three factors of the choice of code—phase or rate—as well as the 0–3 input levels for WM, and the 0–3 stimulus contrast levels all produced significant isolated effects on coding performance, as well as significant interaction effects when tested in a three-factor ANOVA (*p* = 0 for all factors and interactions).

The finding that information coding performance is reduced for the spike rate code in the presence of WM signals can be attributed to the manner in which Poisson-based spike codes depend on baseline firing rate λ_0_. WM inputs are global across the neural field and slightly increase the overall baseline firing rate: Δλ_0_. Likewise, the stimulus in our setup produces another small change Δλ_*s*_ in the average firing rate between the two stimuli-locations *θ* on the neural field. It is a standard result in information theory that Poisson information gain scales as $IG\approx \frac{1}{2}\frac{\Delta \lambda_{s}^{2}}{\lambda_{0}}$. So, the information gain is inversely proportional to baseline firing λ_0_. Thus, any elevation of baseline λ_0_ → λ_0_+Δλ_0_ by WM inputs undercuts discrimination performance: $\frac{\Delta \lambda_{s}^{2}}{\lambda_{0}+\Delta \lambda_{0}}<\frac{\Delta \lambda_{s}^{2}}{\lambda_{0}}$. That is, this change in baseline firing Δλ_0_ due to WM input makes any stimulus driven change Δλ_*s*_ less discriminable through a divisive normalization by λ_0_ (Reynolds and Heeger, 2009).

The MI is a complementary means of assessing coding performance that measures the amount of information shared between the stimulus features over multiple points across the ring and the neural field readout of the phase and count distributions—an MI value of 1 would mean that spike data observations (phase or count) completely determine which stimulus is present. We used eight evenly spaced points across the ring to achieve broad coverage. The MI measure results are qualitatively similar in all respects to the IG measure (Figure 4B). Just as with IG, the MI measure depended significantly on the three factors: choice of code, WM level, and contrast level, as well as significant interaction effects between all factors when tested in a three-factor ANOVA (*p* = 0, for all factors and interactions).

## Discussion

In this article we sought to address how oscillatory changes observed in sensory areas during WM input can possibly enhance oscillatory representations of sensory activity—the phase code—given that mean rate changes associated with WM are weak or nonexistent (Bahmani et al., 2018). To achieve a realistic phase code, we chose a model—our neural mass model—that satisfied the requirement that cortical spikes be only modestly phase locked to the LFP β-oscillation, and the model oscillation must be modulated by WM-like input in a similar fashion to experimental findings. We were able to satisfy to these requirements, and achieve the requisite enhancement of the phase code in response to elevated WM input, by having our model tuned at the point of a certain type of oscillatory instability: a supercritical Hopf point. At this instability point, WM input produces a weak amplitude oscillation continuous with zero. Such weak amplitude oscillations are consistent with experimental findings that cells exhibit weak or intermediate phase locking to the β-band of the LFP, in contrast with large-amplitude oscillations that would necessarily produce strong phase locking and synchrony. The model was derived from, and well-described by, a large-*N* LIF recurrent network of e- and i-cell populations driven by noise input.

By joining a continuum neural mass models into a neural field model across a spatial domain *θ* that represented a stimulus feature, we could preferentially stimulate areas of the domain to create sensory representations in the neural activity (Ben-Yishai et al., 1997; Shriki et al., 2003; Coombes et al., 2014). Both oscillatory and mean rate based representations—the phase and rate codes—were assessed using information theoretic measures as a function of stimulus contrast and WM-input level. The main effect we found was that increasing WM input *increased* phase coding performance while rate coding performance *decreased*. WM also produced an enhancement of contrast gain for the phase code, and a decrease in contrast gain for the rate code. The phase coding performance, owing to the larger information capacity of the continuous phase variable relative to the discrete-valued rate code, was also about two orders of magnitude larger than the rate coding performance.

The mechanism by which WM enhances phase code representations is due to the connection between oscillation amplitude and phase certainty. Specifically, the phase code *p*_*θ*_(ϕ) is a one-to-one transformation with the e-cell firing rate curve *f*_*σ*_(*I*_*e*_(*t*, *θ*)) defined over the oscillation cycle *t* ∈ [0, *T*], at a feature *θ*. This one-to-one map from ϕ ∈ [−π, π] to *t* ∈ [0, *T*] provided a link between oscillation amplitude and phase certainty: WM-driven oscillations with larger amplitude tended to produce spikes in a more concentrated period of time, yielding larger spike phase locking, which in turn, translated to greater probabilistic certainty (reduced variance) in the phase variable. Naturally, reduction of variance makes any two *θ*-value-indexed densities *p*_*θ*_(ϕ) discriminated more readily in the presence of a stimulus.

In contrast to phase coding, the information coding decline of the rate code as a function of WM can be accounted for by the nature of Poisson spiking: information discrimination of small firing rate changes from λ_0_ to λ_0_+Δλ is inversely proportional to the baseline firing λ_0_; therefore, WM input that slightly elevates baseline firing λ_0_ has the effect of undercutting coding performance.

This link between increased oscillation amplitude and increased phase certainty and enhanced information gain was independent of mean firing rate over the oscillation cycle. In our representation of neural activity, the spike rate code is a distinct channel of information from the phase code because the neural activity is readily segmented into the constant and oscillating components of the cycle activity in a Fourier decomposition, respectively: $f_{\sigma }\left(I_{e}\left(t,\theta \right)\right)=\lambda +\left(\overset{\infty }{\underset{n=1}{\sum }}c_{n}e^{i\frac{n\pi }{T}t}+CC\right)$, where λ is the mean firing rate (i.e., the DC mode of the firing rate oscillatory cycle). While we can associate two distinct codes as distinct Fourier features of the neural oscillation, how exactly global WM input can modulate both mean cycle activity λ and the oscillatory features *c*_*n*_ in a coordinated fashion is not fully understood. Specifically, more study is needed to understand how discriminable information is allocated to these distinct channels, as well as how this allocation could be controlled by inputs. In the model we studied here, the overwhelming majority share of discriminable information was allocated to the phase code channel while the mean rate λ exhibited comparably little change in response to stimulus or WM inputs. That our neural field model exhibits a relaxation oscillation offers a plausible explanation for this divergent information allocation: the e-cell rising phase of the cycle is stopped by the i-cell-driven falling phase that partly offsets e-cell firing activity, yielding less average firing rate gain, and so less rate coding performance from an input. This information about an input is still available in the phase variable, encoded the timing of the rise-then-fall phase relative to other parts of the neural field (see Nesse et al., 2021b for a similar perspective on rate coding performance loss through intracellular adaptation in single neuron spike coding).

In our model, we have relied on noise driven spiking of the constituent LIF cells whose average synaptic dynamics is described by our neural mass and field models. Endogenous network fluctuations in the finite-*N* networks were not explicitly accounted for in cell inputs, but clamped to a specific white noise level *σ*. This noise-clamping enabled us to locate supercritical-Hopf oscillatory instabilities in the *I*-*σ* parameter space of the neural mass model. This limiting case, where noise is a fixed exogenous input, is distinct from network models in which mean and co-variability of cells is self-consistently solved for to obtain asynchronous steady states (Moreno-Bote et al., 2008; di Volo et al., 2019; Sanzeni et al., 2020). It is unknown to us if networks derived under such self-consistency constraints can support the emergent oscillations that we studied here.

The results of this modeling study have applications to sensory areas of the brain that are known to receive signals that reflect the content of WM. Consistent with the present modeling results, previous work by our group in areas V4 and MT of the Macaque, has identified that WM signals induce changes in both the oscillatory power, peak-power frequency of LFPs in the α-β band, as well as modulating the occurrence of spikes at certain phases of the LFP oscillation. Also consistent with our results here, it was simultaneously observed that such WM signals to V4-MT elicit small changes to mean firing rate that may not readily account for the observed cognitive benefits produced by WM (Bahmani et al., 2018). The mechanism by which WM modulates phase certainty through amplitude modulations, as well as the prospect of storing vastly more stimulus information in the phase code relative to the rate code, suggests a possible way in which the cognitive benefits of WM are mediated. Note however, that our rate representations of stimuli assumed uncorrelated Poisson spiking that does not account for neuronal correlations that have been shown to be modulated by cognitive states (Cohen and Maunsell, 2009) and it is unknown how such a correlation modulation compares to the phase code modulations we have studied here. Additionally, the potential we have identified for greater sensory information to be contained oscillatory representations begs the question of how such representations are read out by downstream targets. We do not address that question here. Certainly, read out of phase coded information by downstream brain areas is more complicated than the transfer of rate-coded information, but potential mechanisms have been proposed (Comeaux et al., 2023).

## Data availability statement

The datasets presented in this study can be found in online repositories. The names of the repository/repositories and accession number(s) can be found in the article/supplementary material.

## Author contributions

WN designed all analyses, simulations, and wrote the article. KC provided input on text composition, citations, and research design. BN helped administer research design and text composition. All authors contributed to the article and approved the submitted version.
